# The Epidemiology of Injuries in Spanish Rugby Union División de Honor

**DOI:** 10.3390/ijerph19073882

**Published:** 2022-03-24

**Authors:** Roberto Murias-Lozano, Luis Mendía, Francisco Javier San Sebastián-Obregón, Cristian Solís-Mencia, Juan Pablo Hervás-Pérez, Manuel Vicente Garnacho-Castaño, José Luis Maté-Muñoz, Pablo García-Fernández

**Affiliations:** 1 Department of Physiotherapy, Camilo José Cela University, Villafranca del Castillo, 28692 Madrid, Spain; rmurias@ucjc.edu (R.M.-L.); javi-sanse@hotmail.com (F.J.S.S.-O.); jphervas@ucjc.edu (J.P.H.-P.); 2 Spanish Rugby Federation, 28008 Madrid, Spain; 3 Medical Education Department, World Rugby, 10 Pembroke Street Lower, D02 AE93 Dublin, Ireland; lmendia@doctors.org.uk; 4 Independent Medical Practice, Santiago, Chile; csolis.mencia@gmail.com; 5 Nursing Department, Campus Sant Joan de Deu, 08034 Barcelona, Spain; manuelvicente.garnacho@sjd.edu.es; 6 Department of Radiology, Rehabilitation and Physiotherapy, Complutense University of Madrid, 28040 Madrid, Spain; pablga25@ucm.es; 7 IdISSC, Instituto de Investigación Sanitaria del Hospital Clínico San Carlos, 28040 Madrid, Spain

**Keywords:** sports injury prevention, sports epidemiology, sports rehabilitation, injury prevention, professional sport, lower limb, muscle tear

## Abstract

Background: We describe and analyze injury incidence, severity, cause of injury, anatomical location, damaged tissue, injury recurrence, and the time and place at which injuries occur over the course of a season. Methods: An observational, descriptive, prospective, nomothetic, and multidimensional study was conducted during the 2018–2019 season with 258 players of the top semiprofessional rugby league in Spain (División de Honor de Rugby). Data were reported by the clubs’ medical services. Reported time-loss injuries were collected. Results: Overall exposure was 4100 h (137 matches), over 35 weeks of competition. A total of 288 injuries were reported, with three of these leading to withdrawal from the sport. A total average of 35.63 days was lost to injury. Overall time-loss injury incidence was 3.41 injuries/1000 h of exposure. Backs suffered 119 injuries corresponding to 3.80 injuries/1000 h of exposure, whilst forwards suffered 169 injuries with 4.27 injuries/1000 h of exposure. Severe injuries were the most frequent injury type. Conclusions: Outcomes confirm that more injuries take place during competition, with these also being more severe in nature. Contact injuries were most frequently suffered, above all, due to tackling or being tackled.

## 1. Introduction

Rugby is a widely enjoyed sport, with 9.6 million players worldwide and close to 877 million followers. Furthermore, 2.7 million females are currently playing rugby, with participation increasing by 28% since 2017 [1].

Rugby has been played professionally since 1995 and offers diverse formats as a function of the number of players [2]. Spain has experienced exponential growth in both the number of participants and the level of play, and has reached sixteenth in the World Rugby national rankings [3]. Although this places Spain in the category of a Performance Tier 1, the national team has not yet qualified for a Rugby World Cup [4].

Match injury incidence in men’s rugby has previously been reported within the range of 67 to 91 injuries/1000 player-match-hours [5,6,7,8,9,10], and training injury incidence in the range of 1.0 to 3.5 injuries/1000 player-training-hours [5,6,7,8,9]. The match incidence of injury in rugby is higher, but the training incidence of injury is similar compared to football, which reports a match incidence of 36 injuries/1000 player-match-hours and a training incidence of 3.7 injuries/1000 player-training hours [11].

Tackling is one of the differentiating elements of this sport given that it is an event that promotes injuries [6]. In addition to this, other actions specific to Rugby, such as those performed in the ruck, maul, scrum, line-out and collisions often lead to injury [12]. For this reason, rugby is an exponent of catastrophic injuries (2/100,000 per year), with an incidence similar to that seen in American football (2/100,000 per year), but lower than that found in ice hockey (4/100,000 per year) [13].

Tier 1 Rugby Nations have shown that it is a sport that produces multifaceted injuries [14] with different natures between genders [15]. However, it is unknown if the nature and specifics of injuries in Performance Tier 1 Nations are similar to Tier 1 Nations. This raises the question of the relevance of protocols and preventative measures [16] used in professional settings being applied in semi-professional environments.

As far as we are aware, there have been no previously published epidemiological studies of injuries sustained in elite Spanish rugby: the present study, therefore, is the first to conduct an analysis of injuries sustained at the highest level of rugby in Spain. The main aim of the present study was to describe the incidence, severity, nature and causation, of injuries sustained over the course of a full season of elite Spanish rugby.

## 2. Materials and Methods

### 2.1. Design

Study design: Observational, descriptive, prospective, nomothetic, and multidimensional [17].

A data registration sheet, which was based on the international consensus statement proposed by Fuller et al. [18], was used, adding some questions considered to be relevant to our specific study. An injury was considered as “any physical complaint suffered by a player during a match or a training session which impeded that player from fully participating in all training or match activities for more than one day following the day of the injury”. Anthropometric data, left/right dominance, injury type, injury location, affected tissue, injury severity and injury causation factors were recorded [18]. Injury incidence was analyzed separately for matches and training sessions as the number of injuries/1000 h of risk exposure.

The study was approved by the Ethics Committee of the Camilo José Cela University of Madrid. Players (*n* = 258) in eight of the 12 teams involved in the men’s top flight (División de Honor de Rugby) of Spanish rugby agreed to participate in the study and were tracked throughout the 2018/19 season. All clubs were informed about the background and aims of the study. In addition, all players were informed of the nature and characteristics of the study and all players provided written informed consent for their information to be included in the study, in accordance with the Declaration of Helsinki for research with human beings [19].

Medical teams at the clubs recorded injuries each week. Injury diagnosis and data collection were always performed by the same qualified medical practitioner at each club. Players with previous injuries and/or existing injuries were not excluded from the studies, but injuries existing at the start of the study were not included. Players with existing injuries joined the study once they were fully recovered from the injury. Those players who had an unresolved injury, at the end of the season, were followed up until they had fully recovered. Similarly, any player who left their club during the season whilst injured was followed up until the time of full recovery. 

### 2.2. Statistical Analysis

Statistical analysis was carried out using the program SPSS for Windows, version 21 (IBM SPSS: Statistical Package for Social Science. Chicago, IL, USA). The analysis of quantitative variables was expressed through means, standard deviations (SD) and their 95% confidence intervals (CI). Data normality was analyzed using the Kolmogorov–Smirnov test. Mean differences were analyzed using parametric or non-parametric tests, as a function of data normality outcomes. Correlations between quantitative variables were analyzed with Pearson correlation coefficients or Spearman correlation coefficients. Associations between qualitative variables were analyzed through chi-squared comparisons and cross-tabs. *p* < 0.05 was considered to denote statistical significance, with no allowance being made for the number of statistical tests.

## 3. Results

### 3.1. Anthropometrics and Habits

The final study sample consisted of 258 rugby players (backs: 114; forwards: 144) playing in the highest semi-professional league in Spain (División de Honor de rugby). On average, players were aged 25.4 ± 4.6 years, measured 181.6 ± 7.2 cm in height and weighed 94.0 ± 14.1 kg (see Table 1 for full details).

The examination of the association between the quantitative variables related to anthropometric data and training and competition habits did not show a statistically significant relationship with respect to the number of injuries vs. age (*p* = 0.226), weight (*p* = 0.035) or height (*p* = 0.075), or days lost due to injury with respect to age (*p* = 0.499), weight (*p* = 0.351) and height (*p* = 0.084). Only the hours spent on natural grass (*p* = 0.001) and artificial grass (*p* < 0.001) were positively and negatively correlated, respectively, with the number of injuries.

### 3.2. Incidence

Over the 35-week season, 205 team-games, corresponding to a total match exposure of 4100 player-hours (backs: 1.9, forwards: 2.2), and a total training exposure of 66,775 player-hours (backs: 29.3; forwards: 37.4) were registered. In total, 288 injuries were recorded, of which 220 were match injuries (backs: 93; forwards: 127) and 68 were training injuries (backs: 26, forwards: 42).

The overall injury incidence for all players was 4.1 injuries/1000 player-hours (95% CI: 3.7 to 4.6). There was no statistically significant difference (*p* = 0.303) between the overall incidences of injury for backs (3.8, 95% CI: 3.2 to 4.6) and forwards (4.3, 95% CI: 3.7 to 5.0). Match incidence for all players was 53.7 (95% CI: 47.1 to 61.3) injuries/1000 h of exposure. There was no statistically significant difference (*p* = 0.190) between the match incidences for backs (48.6, 95% CI: 39.7 to 59.6) and forwards (58.1; 95% CI: 48.8 to 69.1) Training incidence of injury for all players was 1.0 injuries/1000 h (95% CI: 0.8 to 1.3). There was no statistically significant difference (*p* = 0.358) between the training incidences for backs (0.9, 95% CI: 0.6 to 1.3 and forwards (1.1; 95% CI: 0.8 to 1.5).

### 3.3. Anatomical Location

**Figure 1:**
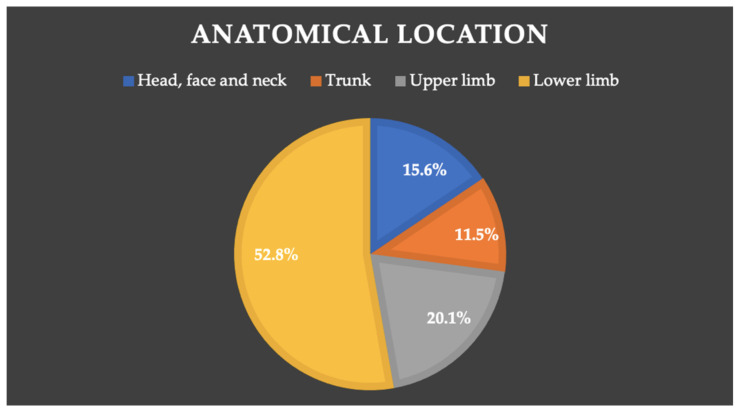
Anatomical location.

The most commonly recorded anatomical location was the lower limb, with 152 (52.8% 95% CI: 46.9% to 58.5%) of the 288 recorded injuries. In the upper limbs, 58 injuries (20.1% 95% CI: 15.4% to 24.8%) were produced, a total of 45 (15.6% 95% CI: 11.4% to 19.8%) injuries affected the head, face and neck, and, finally, 33 (11.5% 95% CI: 7.7% to 15.1%) injuries were suffered by the trunk (Figure 1). Statistically significant differences existed between the anatomical location and injury severity (*p* = 0.045); upper limb injuries were the most severe (48.3 days), whilst trunk injuries had the lowest severity (19.1 days). A statistically significant relationship existed between injury location and the cause of the injury (*p* < 0.001). Specifically, a positive relationship was found between lower limb and trunk and overuse injuries, and between upper limb and head/face/neck and traumatic injuries. Finally, a statistically significant association was also found between trunk injuries and training, whilst upper limb and head/face/neck injuries were related with competition (*p* < 0.001). There were no differences in the days off in training vs. game or forwards vs. backs.

### 3.4. Injured Tissue

**Figure 2:**
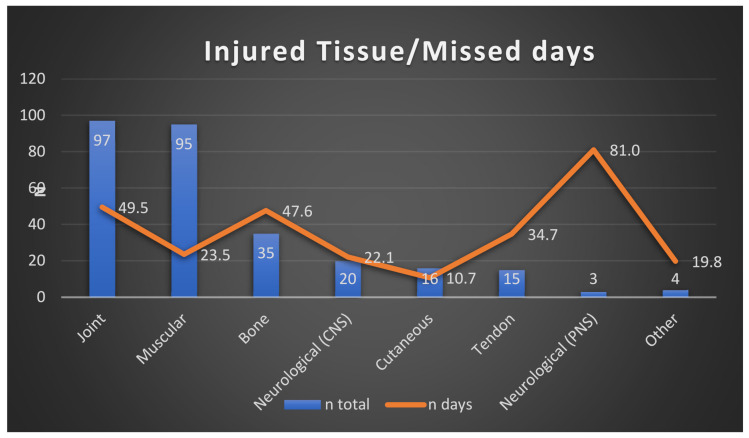
Injured tissue/missed days.

Joint (*n* = 98, 34.0% 95% CI: 28.5% to 39.5%) and muscle (*n* = 95, (33.0% 95% CI: 27.5% to 38.4%) injuries were the most frequent, followed by injuries to bone tissue (*n* = 35, 12.2% 95% CI: 8.3% to 15.9%). The sum of all remaining injuries in neurological and cutaneous tissue and tendons amounted to 60 injuries (20.8%) (see Table 2). Statistically significant differences were observed with regards to injury severity and the affected tissue (*p* = 0.001): injuries affecting the neurons, joints and bones lead to the greatest number of missed days. The most common injuries in training are muscular (64.7%) followed by joints (14.7%), while in the match they are joints (40.0%) followed by muscular (23.2%). Between positions, the forwards are most injured muscularly (37.3%), followed by joints (32.6%), while backs are most injured in joints (36.1%) followed by muscular injury (26.9%). Figure 2 shows the number of missed days of in relation to the injured tissue.

### 3.5. Injury Type

Two main types of injury were found; namely, muscle strains (*n* = 87, 30.2%, 95% CI: 24.8% to 35.5%), and ligament sprains (*n* = 80, 27.8%, 95% CI: 22.5% to 32.9%). Fractures (*n* = 21, 7.3%, 95% CI: 4.2% to 10.3%), concussions (*n* = 20, 6.9%, 95% CI: 3.9% to 9.9%), contusions (*n* = 18, 6.3%, 95% CI: 3.4% to 9.0%) and tendinopathies (*n* = 16, 5.6%, 95% CI: 2.8% to 8.2%) were the next most common types of injury (Table 2). The number of days missed significantly differed with regards to injury type (*p* < 0.001). During training sessions, muscle strains account for 60.3%, while during matches, ligament sprains and muscle strains account for 32.7% and 20.9%, respectively (Table 2).

The most frequent lesion diagnosis was anterior talofibular ligament injury of ankle with 35 lesions (12.2%), followed by hamstring tears 24 (8.3%), and in third place concussions 20 (6.9%) (Table 3).

### 3.6. Severity

Severe and moderate injuries were most common, corresponding to 35.4% (95% CI: 29.8% to 40.9%) and 35.1% (95% CI: 29.5% to 40.6%), respectively. To a lesser extent, 18.4% (95% CI: 13.9% to 22.9%) and 10.07% (95% CI: 6.5% to 13.5%) of injuries were found to be mild and minor, respectively. Three injuries (1.2%) led to the complete withdrawal of players from the sport: peripheral neurological compression, early recurrent anterior shoulder dislocation and rupture of the biceps femoris and semitendinosus conjoint tendon. These three injuries were excluded from all calculations of mean severity.

The average number of days missed due to the 285 recorded injuries was 35.6 days (95% CI: 29.8 to 41.4). A statistically significant difference (*p* = 0.020) was found between the average number of days absence due to injuries sustained during competition (37.5 95% CI: 30.7 to 44.2) and training (28.8 95% CI: 17.5 to 39.9). A statistically significant (*p* = 0.002) association exists between injury severity and the nature (training or match) of exposure. There was a positive relationship between minor injuries and training, with these accounting for 23.8% of all injuries sustained in training.

During matches, forwards tend to suffer moderate injuries (40.3%), the backs, on the other hand, tend to suffer more severe injuries (45.2%).

### 3.7. Injury Mechanism

Contact activities were the most common cause of injury (*n* = 192, 67.4%, 95% CI: 61.9% to 72.8%); 93 (32.6%, 95% CI: 27.1% to 38.0%) injuries were the result of non-contact activities. The nature of these injuries was distributed as follows: making a tackle with 60 injuries (21.1%), being tackled with 49 injuries (17.2%), collisions with 27 injuries (9.5%) and rucking with 25 injuries (8.8%) (Table 4). 

The average severity of contact injuries (35.9 days, 95% CI: 31.4 to 46.6) was significantly (*p* = 0.035) greater than the average severity of non-contact injuries (28.6 days, 95% CI: 20.2 to 36.9). Most of the injuries occurred without contact (69.1%). In matches, injuries are caused by tackling (24.1%) and being tackled (21.4%).

### 3.8. Injury Cause

The most common cause of injury was trauma, with 181 injuries (63.5%, 95% CI: 57.9 to 69.1), whilst 104 injuries (36.5%, 95% CI: 30.8% to 42.0) were due to overuse. The average severity of traumatic injuries (40.0 days, 95% CI: 32.1 to 47.8) was significantly (*p* = 0.003) greater than that of overuse injuries (28.1 days 95% CI: 19.9 to 36.1).

Statistically significant differences (*p* = 0.010) also emerged as a function of the cause of the injury, highlighting an association between injury recurrence and overuse.

Playing position was also significantly associated (*p* = 0.020) with injury causation, with traumatic injuries more likely to be sustained by backs and overuse injuries by forwards. The percentages are reversed if we observe the frequencies in training (70.6% trauma, 29.4% overuse) and in matches (74.1% overuse, 25.9% trauma).

### 3.9. Place of the Injury

Statistically significant differences existed (*p* = 0.049) between the mean number of days missed following injuries sustained on natural grass (*n* = 202, 37.9 days, 95% CI: 30.7 to 44.9) and artificial turf (*n* = 83, 30.2 days, 95% CI: 20.3 to 40.1).

Playing position was a statistically significant factor with regards to the moment of injury (*p* = 0.039), with those playing in the front row more likely to be injured during training and those in the back row being more commonly injured during matches.

### 3.10. Time during a Match

Thirty-two injuries (14.5%, 95% CI: 10.0% to 19.3%) were sustained during the first quarter (forwards: 13; backs: 19) of matches, 49 (22.3%, 95% CI: 16.7% to 27.7%) during the second quarter (forwards: 26; backs: 23), 77 (35.0%, 95% CI: 28.4% to 40.9%) during the third quarter (forwards: 52; backs: 25) and 62 (28.2%, 95% CI: 22.5% to 34.3%) during the fourth quarter (forwards: 36; backs: 26).

### 3.11. Injury Recurrence

A total of 24.7% (95% CI: 19.6% to 29.6%) of injuries sustained were recurrent injuries. No statistically significant differences existed between injury severity and index and recurrences (*p* = 0.274). Nonetheless, a statistically significant positive association did exist between recurrence and injuries evaluated as being minor. In contrast, injuries classified as severe were associated with being one-time injuries (i.e., non-recurrence) (*p* = 0.048). In addition, a statistically significant relationship was found between recurrence and injury location (*p* = 0.027), with trunk injuries being more likely to reoccur.

## 4. Discussion

The present study sought to examine injuries and the pattern of injuries in top flight rugby in Spain.

On average, players were aged 25.4 ± 4.6 years, weighed 83.2 ± 8.8 kg and measured 181.6 ± 7.2 cm in height. This coincides with other studies in which the average age was between 24.8 and 25.3 years [20,21,22] and average height was between 179 and 181.5 cm [10,23,24]. In contrast, some other previously conducted studies reported older average ages of examined players, with ages ranging between 27.4 and 27.6 years [5,7,8]. Furthermore, average height and weight have also been reported to be greater, with between 185.1 and 187.0 cm [5,7,8,20,21] and 100.0–104.1 kg [5,7,8,20,21,22], respectively, being reported. These differences in anthropometric values may be due to the fact that these studies were conducted in Tier 1 countries, in which anthropometric values tend to be greater to those seen in Mediterranean countries [25]. Furthermore, most of these studies were carried out in countries either where rugby has been a professional sport for many years, for instance in Australia [20] and England [22], or collected data related to top level competitions [5,7,8]. For these reasons, players would be expected to be taller and heavier [26].

Total incidence was 4.1 injuries/1000 h of exposure, corresponding to 1.02 injuries/1000 h of exposure during training and 53.7 injuries/1000 h of exposure during competition. These data are similar to those produced in similar studies conducted in lower level rugby [10,23], but the incidence during competition was significantly lower than those reported for professional clubs and international rugby tournaments (81 to 138 injuries/1000 h of competition) [5,7,8,24,25]. This difference is potentially due to less rest time between games in international tournaments and the greater level of physicality in elite games, which leads to higher intensity and more frequent contact events. In contrast, these same studies reported similar incidence values to the present study with regards to training, (1.0 to 3.5 injuries/1000 h of exposure) [5,7,8,9,21,24,27].

Forwards were injured 169 times (4.3 injuries/1000 h of overall exposure), whilst backs sustained 119 (3.8 injuries/1000 h of overall exposure) injuries. A number of previous studies have reported similar data [5,7,10,21,23]. Similarly, there were no significant differences in injury severity for backs or forwards.

The most commonly injured tissue pertained to the joints, accounting for 33.7% of injuries, followed by muscular tissue or tendons with 33.0%. Injury incidence in joints and muscles was 21.7 and 13.2 injuries/1000 h of overall exposure, respectively. These data are similar to those produced in the majority of previously conducted studies. This reveals a predominance of joint and muscle injuries both with regards to incidence [5,7,28] and prevalence [8,10,21,23].

The most common injury types were muscle rupture, strains or tears, accounting for 30.2% of injuries, followed by sprains or ligament injuries which accounted for 27.8%. Other studies have presented significantly fewer injuries, ranging from 14% to 24.1% [7,10,23,24]. This difference may be due to the greater time spent in weekly training and/or gym sessions in the present study, in addition to the more professional setting given that the aforementioned studies with lower injury rates were conducted in other professional leagues or World Cups.

The average number of days missed due to injury was 35.6 days in the present study. Similar figures were reported by Fuller et al. [8] and Whitehouse et al. [20] with 29.8 and 37.4 missed days, respectively. Nonetheless, other studies have reported lower values [5,7,10]. This difference may again be due to the fact that these other studies relate to international competitions where there is often the need for players to return to competition more quickly. 

Serious injuries were the most common, followed by moderately serious injuries. Previous studies, such as those conducted by Solís-Mencia et al. [24] and Schneiders et al., [23] present similar findings. Nonetheless, in other studies, light and minor injuries predominated, accounting for 50–64% [10,27,28] of injuries. These differences may be due to anthropometric issues of the players included in these studies [25], which, possibly, indicate a physical makeup that is more adapted to the mechanical demands of play and more resistant to impact by other players.

The majority of injuries were produced as a result of contact (67.4%). Strong consensus exists within the published literature that the most frequent injury mechanism in rugby is contact [5,7,8,10,20,21,23,24,27,28].

The highest risk action was tackling, accounting for 60 injuries (31.2%). In this regard, no consensus exists given that a number of authors point to tackling as being the most injurious action [21,23], whilst others indicate that being tackled leads to the most injuries [5,7,8,20,24]. Although no consensus exists between studies, it is clear that tackles (tackling and being tackled) are the most common injury mechanism in all studies.

The majority of injuries occurred in the second half of matches (63.2%), compared to the first half (36.8%). The third quarter was responsible for the highest injury incidence, accounting for 35.0% of all injuries, whilst the final quarter led to 28.2% of injuries. These data are similar to those reported in other studies [8,21,23,24,28], which also reported most injuries to be sustained during the third quarter, specifically, between 27.1% and 44.8% of injuries. This may be due to the onset of player fatigue at this time, leading to increases in players’ physical and psychological stress during the second part of matches and just after the break.

The main strength of the present study is that it is the first study carried out in Spain on injuries in the highest category of rugby, describing the characteristics, incidence and severity, with the same methodology used in studies carried out in other countries with higher level competitions. In this way, it increases our knowledge about injuries that occur during competition and training, and can be a valuable tool for implementing preventive measures to increase safety and reduce the number of days off work suffered by athletes.

The present study has some limitations that we consider necessary to point out. The first is that not all the players in the category decided to participate in the study, although a significant percentage did. Another limitation of the study is that although an adequate methodology was followed for recording the data, as they were recorded by the different medical services of the clubs, there may be slight differences. In addition, it was not possible to follow the players during training camps with the respective national teams.

## 5. Conclusions

The lack of epidemiological studies on rugby in Spain makes it necessary to study the sports habits and injuries produced during the practice of this sport in order to contribute to its prevention. In the top flight of Spanish rugby, overall injury incidence was 4.1 injuries/1000 overall hours of exposure, with 53.7 injuries/1000 h of game exposure and 1.0 injuries/1000 h during training. More injuries were sustained during competition, with these injuries also being more serious than those picked up during training. Contact was the most common cause of injury, above all due to tackling or being tackled. With regards to playing position, forwards were the most commonly injured players, and injuries most often occurred during the final two quarters of matches.

## Figures and Tables

**Table 1:** Anthropometric data and exposure.

	Full Sample		Backs		Forwards	
	(*n* = 258)		(*n* = 114)		(*n* = 144)	
	Mean	SD	Mean	SD	Mean	SD
Weight (kg)	94.0	14.2	83.2	8.8	102.5	11.6
Height (cm)	181.6	7.2	178.5	5.9	184.1	7.1
Age (years)	25.4	4.6	24.3	3.8	26.2	5.0
Years playing rugby	12.4	5.5	12.3	5.1	12.4	5.8
Training days/week	4.0	0.7	4.0	0.7	4.0	0.8
Training hours/day	2.4	0.5	2.4	0.5	2.4	0.5
Hours spent training in the gym	3.5	1.6	3.5	1.6	3.6	1.5
Hours spent training on natural turf	5.0	2.7	5.0	2.8	5.0	2.7
Hours spent training on artificial turf	0.9	1.6	1.0	1.7	0.9	1.6
N previous injuries	1.6	1.3	1.6	1.3	1.6	1.4

**Table 2:** Injured tissues and injury type.

Injured Tissue	Injury Type	*n* Injury Type	% Injury Type	*n* Total	% Total
Joint				98	34.0%
	Sprain/Ligament injury	80	27.8%		
	Dislocation/Subluxation	14	4.7%		
	Meniscus, cartilage or disk injury	2	0.7%		
	Bruises, bumps or contusions	2	0.7%		
Muscular				95	33.0%
	Rupture, strain, tear or cramp	87	30.2%		
	Bruises, bumps or contusions	8	2.8%		
Bone				35	12.1%
	Fracture	21	7.3%		
	Other bone injuries	8	2.8%		
	Bruises, bumps or contusions	6	2.1%		
Neurological (CNS)				20	6.9%
	Concussion	20	6.9%		
Cutaneous				16	5.6%
	Laceration, cut or wound	15	5.2%		
	Abrasion or severe scrape	1	0.3%		
Tendon				16	5.6%
	Tendinopathy, tendon rupture or bursitis	16	5.6%		
Neurological (PNS)				4	1.4%
	Spinal compression	3	1.0%		
	Peripheral nerve injury	1	0.3%		
Other				4	1.4%
	Dental injury	2	0.7%		
	Bruises, bumps or contusions	2	0.7%		
				288	100.00%

**Table 3:** Most frequent injury diagnosis.

	*n* (%)	Injury Incidence	IC 95%
Anterior talofibular ligament injury (Ankle)	35 (12.2%)	0.5/1000 h	(0.3–0.7)
Hamstrings tears	24 (8.3%)	0.3/1000 h	(0.2–0.5)
Concussion	20 (6.9%)	0.3/1000 h	(0.2–04)
Low back pain	19 (6.6%)	0.3/1000 h	(0.1–0.4)
Calf tears	17 (5.9%)	0.2/1000 h	(0.1–0.4)
Medial collateral ligament injury (Knee)	15 (5.2%)	0.2/1000 h	(0.1–0.3)
Quadriceps tears	14 (4.9%)	0.2/1000 h	(0.1–0.3)
Acromioclavicular joint injury (Shoulder)	13 (4.5%)	0.2/1000 h	(0.1–0.3)
Glenohumeral joint luxation (Shoulder)	8 (2.8%)	0.1/1000 h	(0.0–0.1)
Fracture of the nose	5 (1.7%)	0.1/1000 h	(0.0–0.1)
Anterior cruciate ligament injury (Knee)	5 (1.7%)	0.1/1000 h	(0.0–0.1)

**Table 4:** Injury mechanism and missed days.

	*n*	Mean (Days)	SD	Minimum	Maximum
Without contact	93	28.6	40.5	2	231
Being tackled	49	57.5	70.7	4	266
Tackling	60	38.7	55.0	2	308
Maul	2	52.5	23.3	36	69
Ruck	25	27.4	37.3	2	174
Line-out	6	26.5	10.4	14	41
Scrum	12	42.6	59.3	2	209
Collision	27	27.4	28.9	2	90
Other	11	14.3	10.0	4	37
Total	285	35.6	49.8	2	308

## Data Availability

Not applicable.
